# Assessment of the Representation of Civil Servants as the General Population in Terms of Health Checkup Data

**DOI:** 10.7759/cureus.96701

**Published:** 2025-11-12

**Authors:** Yoshiharu Fukuda, Kumi Sugimoto, Haruka Nakagawa, Takuya Yamada

**Affiliations:** 1 Graduate School of Public Health, Teikyo University, Tokyo, JPN

**Keywords:** civil servants, geographic variation, health checkups, national database (ndb), real-world data (rwd)

## Abstract

Purpose: Large-scale datasets of government workers have been increasingly used to generate robust epidemiological evidence. However, it remains unclear whether this specific occupational group is representative of the general population in health-related data. This study aimed to examine whether health checkup data from government workers are representative of the general population.

Methods: We analyzed health checkup data from the Mutual Aid Association (MAA) for local government employees and from the National Database of Japan (NDB) across 46 prefectures. Sex-specific and age-adjusted prevalences (AAPs) of health examination abnormalities, medication use, and health behaviors were calculated for each prefecture. Correlation coefficients between the two populations were then determined to evaluate the similarity of regional patterns.

Results: Among men, all health examination abnormalities were significantly less prevalent in MAA than in NDB. Among women, the prevalence was also lower in MAA than in NDB, except for HbA1c and liver dysfunction. The MAA population showed significantly lower medication use for hypertension and diabetes mellitus, but higher use for dyslipidemia. Unfavorable health behaviors such as smoking, weight gain, and habitual breakfast skipping were less common in MAA, whereas favorable behaviors such as regular exercise and physical activity were more frequent among men. Correlation coefficients of AAPs ranged from 0.36 to 0.88 for examination abnormalities, 0.21 to 0.80 for medication use, and 0.35 to 0.73 for health behaviors.

Conclusion: Significant positive correlations were observed between MAA and NDB in the prevalence of health examination abnormalities and health behaviors, indicating that regional health patterns among government workers closely mirror those of the general population. These findings suggest that health data from government workers can serve as a valuable resource for assessing regional differences in population health in Japan.

## Introduction

Real-world data (RWD) and big data have been increasingly utilized in epidemiological and health policy research [1,2]. The accumulation of health care and health checkup data, combined with the ongoing digital transformation in health systems, has greatly facilitated such research. Consequently, a growing number of epidemiological studies using RWD and big data, such as retrospective cohort analyses and quasi-experimental studies, have been published in recent years [3-6].

In Japan, the use of health insurance databases by public insurers, local and national governments, and academic institutions has been actively promoted under the national “Data Health” initiative [7]. These databases often include longitudinal information on health checkups, medical care utilization, and work-related variables, particularly among occupational groups and insured populations. As a result, numerous studies based on data from specific occupational cohorts and insurers have been conducted [4,8-10].

Government workers represent one such occupational group frequently used in epidemiological research. Previous studies have collected data from government workers through prospective cohort surveys [11-13]. Because this population is characterized by stable employment, it is relatively easy to obtain complete follow-up information on their health checkups and medical records. This stability makes government workers an attractive population for longitudinal analyses. We have also conducted previous analyses using health data from local government workers in Japan [14-16]. However, an important limitation of such studies is that it remains unclear whether findings derived from government workers can be generalized to the broader population [16].

Therefore, the present study aimed to examine whether health checkup data from government workers are representative of the general population, comparing the results of health checkups between prefectural populations of government workers registered in the Mutual Aid Association (MAA) database and those of the general population.

## Materials and methods

Data and study subjects

We used data from the specific health checkups obtained in 2019 of subjects registered in the Mutual Aid Association of Prefectural Government Personnel (MAA) [17]. MAA covers local government workers and their dependents in all prefectures of Japan (N = 46), excluding Tokyo. The specified health checkups are mandatory for public health insurers nationwide for people aged 40 to 74 years [18,19]. The items and methods of health examination, as well as the survey questions, are standardized, and health guidance to prevent metabolic syndrome is provided to the subjects based on the results of the health checkups. The health checkups data was obtained from the headquarters of MAA, which clears and manages health checkups and insurance claim data. Of all the data, we used the data of subjects aged 40 to 59 years, since people over the age of 60 years are generally retired and the number is limited.

The National Database of Health Insurance Claims and Specific Health Checkups of Japan (NDB) is a nationwide database collected from medical receipts and health checkups conducted by public health insurers and summarized by the Ministry of Health, Labour and Welfare of Japan [20]. The aggregated values are published each year by the website and can be freely accessed and used [20]. The prefecture to which individual data belong is identified by the postal code of the insured person included in the data. The number of people in each five-year age group is published. In this survey, we obtained and used the 2019 NDB data from the website [20]. As with the MAA, we used the data of subjects aged 40 to 59 years.

The cutoff values for abnormal results of the health examination were according to the national program of the specific health checkups [18]: waist circumference (WC) ≥85 cm for men and ≥90 cm for women, Body Mass Index (BMI) ≥25 kg/m^2^, systolic blood pressure (SBP) ≥140 mmHg, diastolic blood pressure (DBP) ≥85 mmHg, serum low-density lipoprotein cholesterol (LDL-C) ≥160 mg/dl, serum triglycerides (TG) ≥150 mg/dl, hemoglobin A1c (HbA1c) ≥5.6%, serum alanine aminotransferase (ALT) ≥31 IU/dl, serum aspartate aminotransferase (AST) ≥31 IU/dl, and serum gamma-glutamyl transferase (GGT) ≥51 IU/dl.

The items of medication use and health behaviors were selected from the responses to the questionnaire included in the specific health checkups. The medication use for hypertension, diabetes mellitus, and dyslipidemia was reviewed. The health behaviors include smoking, weight gain, habitual exercise, habitual physical activity, fast walking, fast eating, late dinner, habitual snacks and beverage consumption, habitual skipping of breakfast, daily drinking and sufficient sleep. The definitions are shown in Table 1.

**Table 1:** Definitions of health behaviors: questions and responses in the health checkups

Health behavior	Question	Response
Smoking	Are you a regular smoker?	Yes
Weight gain	Have you gained at least 10 kilograms since you were 20 years old?	Yes
Habitual exercise	Have you exercised moderately (enough to produce a light sweat) for 30 minutes or more at least twice a week for at least one year?	Yes
Habitual physical activity	Do you walk or engage in an equivalent level of physical activity for at least one hour a day as part of your daily routine?	Yes
Fast walking	Do you walk faster than someone roughly of the same age and sex?	Yes
Fast eating	How quickly do you eat compared to others?	Faster
Late dinner	Do you eat less than two hours before going to bed three or more times a week?	Yes
Habitual snacks and beverage consumption	How often do you eat snacks or drink sweet beverages between meals?	Little
Habitual skipping of breakfast	Do you skip breakfast three or more times a week?	Yes
Daily drinking	How often do you drink alcoholic beverages?	Every day
Sufficient sleep	Do you feel refreshed after a night’s sleep?	Yes

Analysis

There were missing values for some items. Since the missing values for the health examination by prefecture accounted for less than 1%, all the data were used. For the questionnaire items, data from prefectures (Akita, Aichi, and Kyoto) with 50% or more missing values for items that were not mandatory to report were not included in the analysis.

The age-adjusted prevalences (AAPs) of abnormalities in the health examination items, medication use and health behaviors were calculated by the direct method using the Japanese model population of 2015, which is commonly used for calculation of age-adjusted mortality rate in Japan [21]. Since the Shapiro-Wilk test and the Kolmogorov-Smirnov test indicated that several variables did not follow a normal distribution, all statistical analyses were performed using nonparametric tests. AAPs of MAA were not and NDB were compared using the Wilcoxon signed-rank test. The Spearman’s rank correlation coefficients of AAPs between MAA and NDB were calculated. A significance level of 0.05 was employed. Microsoft Excel (Microsoft Corporation, Redmond, USA) was used for the AAP calculation and IBM SPSS Statistics for Windows, Version 29 (Released 2023; IBM Corp., Armonk, New York, United States) was used for the statistical tests.

## Results

The mean number of subjects per prefecture in the MMA was 2,666.5 (SD = 1,155.3) for men, ranging from 1,359 to 7,920, and 1,801.3 (SD = 849.5) for women, ranging from 748 to 4,739, based on abdominal circumference data.

Table 2 presents AAPs of health check-ups in the MDB and MMA. Among men, all health examination abnormalities were significantly less frequent in the MMA than in the NDB. Among women, except for HbA1c and liver dysfunction, the prevalence of abnormal findings was also lower in the MMA compared with the NDB.

**Table 2:** Comparison of the age-adjusted prevalence of abnormalities in health examination, medication use, and health behaviors in the National Database (NDB) and Mutual Aid Association (MAA) T-value and P were estimated by the Wilcoxon signed-rank test

	Male				Female			
	NDB	MAA	T-value	p	NDB	MAA	T-value	p
	Mean ± SD	Mean ± SD			Mean ± SD	Mean ± SD		
Health examination								
Waist circumference: >=85 cm (Men), >= 90 cm (Women)	49.3 ± 2.5	43.6 ± 4.7	-5.9	<0.01	14.1 ± 1.8	12.7 ± 2.0	-4.9	<0.01
Body mass index: >=25 kg/m^2^	37.8 ± 2.7	32.8 ± 3.8	-5.9	<0.01	21.3 ± 2.6	19.1 ± 2.7	-5.4	<0.01
Systolic blood pressure: >=140 mmHg	16.8 ± 1.7	12.7 ± 2.8	-5.6	<0.01	10.1 ± 1.3	7.6 ± 1.5	-5.8	<0.01
Diastolic blood pressure: >=85 mmHg	31.5 ± 2.9	27.7 ± 5.3	-5.0	<0.01	14.7 ± 1.8	11.9 ± 2.2	-5.5	<0.01
Serum LDL-cholesterol: >=140 mg/dl	32.3 ± 1.4	29.5 ±2.8	-5.5	<0.01	27.8 ± 1.1	26.6 ± 1.9	-4.6	<0.01
Serum triglycerides: >=150 mg/dl	28.9 ± 1.9	23.9 ± 3.3	-5.8	<0.01	9.4 ± 1.0	8.2 ± 1.7	-5.0	<0.01
Hemoglobin A1c: ≥5.6%	45.4 ± 4.3	43.0 ± 9.1	-1.9	0.06	40.3 ± 4.8	40.7 ± 8.3	0.9	0.39
Serum alanine aminotransferase (ALT): >=31 IU/dl	17.5 ± 1.6	15.7 ± 2.0	-5.3	<0.01	6.5 ± 0.6	6.4 ± 0.8	-0.5	0.59
Serum aspartate aminotransferase (AST): >=31 IU/dl	32.0 ± 2.0	28.8 ±2.5	-5.9	<0.01	9.1 ± 1.0	9.2 ± 1.2	0.2	0.77
Serum gamma-glutamyltransferase (GGT): >=51 IU/dl	31.6 ± 3.1	27.7 ± 3.3	-5.9	<0.01	8.5 ± 1.1	8.4 ± 1.3	-1.3	0.20
Medication use								
Hypertension	16.2 ± 1.8	15.9 ± 2.3	-1.4	0.17	8.9 ± 1.2	8.3 ± 1.2	-3.5	<0.01
Diabetes mellitus	5.3 ± 0.5	4.6 ± 0.8	-5.2	<0.01	1.9 ± 0.3	1.6 ± 0.4	-3.8	<0.01
Dyslipidemia	9.5 ± 1.0	11.3 ± 1.8	5.8	<0.01	6.5 ± 0.7	7.2 ± 1.3	4.0	<0.01
Health behaviors								
Smoking	38.9 ± 3.3	19.4 ± 3.1	-5.8	<0.01	19.4 ± 3.1	3.0 ± 1.2	-5.8	<0.01
Weight gain	49.0 ± 2.3	43.3 ±3.6	-5.7	<0.01	29.5 ± 2.9	25.9 ± 3.5	-5.6	<0.01
Habitual exercise	23.0 ± 2.4	27.7 ± 4.1	5.0	<0.01	16.1 ± 2.4	16.0 ± 2.1	0.5	0.60
Habitual physical activity	36.9 ± 3.5	29.9 ± 6.8	-5.3	<0.01	36.7 ± 5.2	34.1 ± 7.4	-3.3	<0.01
Fast walking	45.3 ± 3.4	45.6 ± 6.7	1.8	0.80	39.5 ± 3.7	40.3 ± 5.0	2.5	0.01
Fast eating	38.9 ±1.7	39.9 ± 5.1	1.3	0.21	29.3 ± 1.5	30.8 ± 4.2	2.4	0.02
Late dinner	40.9 ± 3.1	32.1 ± 4.0	-5.7	<0.01	23.3 ± 2.2	25.9 ± 5.4	3.2	<0.01
Habitual snacks and sweets consumption	30.3 ± 2.1	30.4 ± 5.5	0.9	0.37	12.1 ± 1.6	12.2 ± 4.9	-2.0	0.05
Habitual skipping of breakfast	25.8 ± 2.9	12.7 ± 2.7	-5.7	<0.01	15.4 ± 2.8	10.2 ± 2.5	-5.7	<0.01
Daily drinking	36.3 ± 2.9	29.6 ± 5.1	-4.9	<0.01	14.6 ± 1.8	10.2 ± 2.6	-1.1	0.27
Sufficient sleep	62.0 ± 3.4	64.2 ± 10.8	3.5	<0.01	57.9 ± 2.6	57.2 ± 7.8	0.0	0.99

Regarding medication use, MMA had significantly lower prevalence of medication use for hypertension and diabetes mellitus, but a higher prevalence for dyslipidemia.

With respect to health behaviors, some indicators differed significantly between the two populations, while others did not. Unfavorable behaviors, such as smoking, weight gain, and habitual skipping of breakfast, were less common in MMA. Conversely, favorable behaviors such as habitual exercise and regular physical activity were less prevalent among men in MMA.

Table 3 shows the correlation coefficients of AAPs between NDB and MMA. In men, the prevalence of most abnormalities and questionnaire responses was strongly correlated between the two datasets. A similar trend was observed in women, although the correlations were generally weaker.

**Table 3:** Correlation coefficients (Spearman) of prevalences of abnormalities in health examination, medication use and health behaviors between the National Database (NDB) and Mutual Aid Association (MAA)

	Male		Female	
	Coefficients	p-value	Coefficients	p-value
Health examination				
Waist circumference: >=85 cm (Men), >=90 cm (Women)	0.74	<0.01	0.55	<0.01
Body mass index: >=25 kg/m^2^	0.81	<0.01	0.66	<0.01
Systolic blood pressure: >=140 mmHg	0.50	<0.01	0.57	<0.01
Diastolic blood pressure: >=85 mmHg	0.53	<0.01	0.70	<0.01
Serum LDL-cholesterol: >=140 mg/dl	0.37	0.01	0.64	<0.01
Serum triglycerides: >=150mg/dl	0.71	<0.01	0.52	<0.01
Hemoglobin A1c: ≥5.6%	0.53	<0.01	0.56	<0.01
Serum alanine aminotransferase (ALT): >=31 IU/dl	0.60	<0.01	0.28	0.06
Serum aspartate aminotransferase (AST): >=31 IU/dl	0.67	<0.01	0.29	0.06
Serum gamma-glutamyltransferase (GGT): >=51 IU/dl	0.86	<0.01	0.63	<0.01
Medication use				
Hypertension	0.74	<0.01	0.54	<0.01
Diabetes mellitus	0.61	<0.01	0.18	0.22
Dyslipidemia	0.67	<0.01	0.58	<0.01
Health behaviors				
Smoking	0.66	<0.01	0.49	<0.01
Weight gain	0.57	<0.01	0.71	<0.01
Habitual exercise	0.59	<0.01	0.49	<0.01
Habitual physical activity	0.54	<0.01	0.76	<0.01
Fast walking	0.68	<0.01	0.70	<0.01
Fast eating	0.67	<0.01	0.46	<0.01
Late dinner	0.29	0.06	0.47	<0.01
Habitual snacks and sweets consumption	0.53	<0.01	0.59	<0.01
Habitual skipping of breakfast	0.69	<0.01	0.54	<0.01
Daily drinking	0.70	<0.01	0.78	<0.01
Sufficient sleep	0.51	<0.01	0.49	<0.01

**Figure 1:**
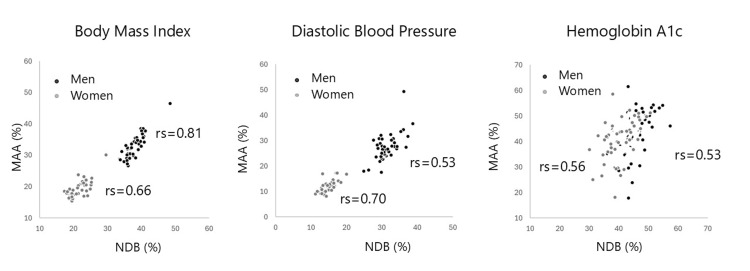
Scatter diagrams of age-adjusted prevalence of abnormalities in selected health examinations between MAA and NDB rs: Spearman’s rank correlation coefficients; NDB: National Database; MAA: Mutual Aid Association

**Figure 2:**
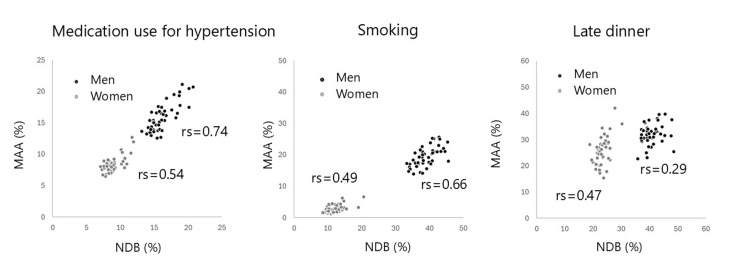
Scatter diagrams of age-adjusted prevalence of questionnaire response in selected items between MAA and NDB rs: Spearman’s rank correlation coefficients; NDB: National Database; MAA: Mutual Aid Association

Figure 1 illustrates scatter plots of health examination abnormalities. Strong correlations were observed for some variables, such as BMI and SBP, whereas others, such as HbA1c, showed weaker correlation. Figure 2 displays scatter plots of questionnaire responses; correlations were strong for medication use for hypertension and for smoking, but weak for late dinner among men.

## Discussion

The results of the health checkups demonstrated moderate to strong positive correlations between the two populations, with coefficients ranging from 0.37 to 0.86 in men and from 0.28 to 0.70 in women. These significant correlations between MAA and NDB populations suggest that regional variations in the prevalence of health abnormalities and questionnaire responses among government workers reflect corresponding regional variations in the general population. The weaker correlations observed for liver function abnormalities in women may be explained by the low prevalence of such abnormalities, resulting in statistical instability.

Overall, the prevalence of most health examination abnormalities was lower in MAA than in NDB. This finding is consistent with previous reports indicating that government workers tend to exhibit fewer health abnormalities than other occupational groups [22]. These results collectively suggest that government workers are generally healthier than the general population.

Similarly, strong correlations were observed in the rates of medication use between the two populations, indicating that regions with higher medication use among government workers also showed higher rates in the general population. However, no significant correlation was observed for the use of antidiabetic medication among women, likely due to the low average prevalence (<2%) and the resulting statistical instability.

The overall prevalence of antihypertensive and antidiabetic medication use was lower in MAA than in NDB, again suggesting better health status among government workers. Conversely, the prevalence of dyslipidemia medication use was higher in MAA than in NDB. Because decisions to initiate treatment for dyslipidemia often depend on individual adherence and treatment intentions [23], it is plausible that government workers are more likely to seek treatment for dyslipidemia even when their overall health status is comparable to that of the general population.

Government workers also demonstrated more favorable health behaviors, including lower rates of smoking, weight gain, daily drinking, and habitual breakfast skipping, as well as higher rates of regular exercise and physical activity. These findings are consistent with prior evidence that government and white-collar employees tend to exhibit healthier lifestyle behaviors than other occupational groups [22,24].

Several factors may explain this tendency. First, government workers generally have stable employment and income, and higher socioeconomic status has been strongly associated with healthier behaviors-such as nonsmoking, greater physical activity, and better dietary habits [25-27], as well as better health outcomes, including lower cardiovascular disease risk [28]. Second, the work environments of government workers tend to be supportive of health; for example, public facilities are required by the Health Promotion Act to be smoke-free. Third, health management systems for government workers are well established, as reflected in their high health checkup participation rate (85.8% in MAA, compared with 55.6% in the general population) [29].

Strong correlations (r > 0.5) were also observed between MAA and NDB for several health behaviors, suggesting that geographic variations in health behaviors among government workers mirror those in the general population. Previous studies have documented regional differences in smoking, drinking, diet, and physical activity in Japan [30]. These variations likely reflect shared sociocultural and environmental determinants that influence both government workers and the general population. Such geographic patterns in health behaviors may, in turn, explain regional differences in health outcomes observed in both populations.

Collectively, the similarity in geographic patterns of health status and health behaviors between government workers and the general population indicates the utility of analyzing data from government worker cohorts. Such data may serve as a useful proxy for evaluating regional health differences and trends in the general population. Thus, databases of government workers could contribute to monitoring and understanding regional disparities in health and healthcare in Japan.

This study has several limitations. First, data from Tokyo, the largest prefecture in Japan, were not included. The population of Tokyo has the largest population with diversity, and thus the representativeness of the government workers may be limited. Nevertheless, other major metropolitan areas, such as Osaka, Kanagawa, and Aichi prefectures, were represented, and the absence of a single region is unlikely to have materially affected the overall findings. Second, participation rates in health checkups differ between government workers and the general population, being substantially higher among the former [30]. Therefore, while MAA data may adequately represent government workers, NDB data might not fully represent the entire general population. Third, caution is required when extrapolating the results of this study beyond Japan, due to differences in social systems between countries. Fourth, MAA data included that of dependents of the government workers. Therefore, not only household but also individual characteristics of the dependents might influence the results. In addition, some statistical issues should be acknowledged. The potential confounding factors, such as socioeconomic status, in the comparison of two populations were not considered. Moreover, we have made numerous comparisons; thus, there is a possibility of alpha error.

In summary, the prevalence of health examination abnormalities was lower, and the proportion of favorable health behaviors was higher, among government workers compared with the general population. Moreover, geographic variations in health outcomes and behaviors were similar between the two groups. Although data from government workers are not entirely representative of those from the general population, they can still serve as a valuable resource for assessing regional differences in health status and behaviors across Japan.

## Conclusions

This study showed positive correlations of the prevalence of abnormalities and health behaviors between government workers and the general population, and also a better health status in government workers. Although the data obtained for government workers were not absolutely representative of those of the general population, they could still be considered as being effective for examining regional differences in the health status and health behaviors of the general population.

## References

[1] Yotsumoto H, Kaneko H, Itoh H (2021). Promoting analysis of real-world data: prospects for preventive cardiology in Japan. Glob Health Med.

[2] Mallappallil M, Sabu J, Gruessner A, Salifu M (2020). A review of big data and medical research. SAGE Open Med.

[3] Hiramatsu K, Barrett A, Miyata Y (2021). Current status, challenges, and future perspectives of real-world data and real-world evidence in Japan. Drugs Real World Outcomes.

[4] Kimura Y, Jo T, Matsui H, Yasunaga H (2024). Clinical research using real-world data: a narrative review. Respir Investig.

[5] Tonegawa-Kuji R, Kanaoka K, Iwanaga Y (2023). Current status of real-world big data research in the cardiovascular field in Japan. J Cardiol.

[6] Imaizumi T, Toda T, Maekawa M (2022). Identifying high-risk population of depression: association between metabolic syndrome and depression using a health checkup and claims database. Sci Rep.

[7] (2025). Ministry of Health. Labour and Welfare. Data Health. https://www.mhlw.go.jp/stf/seisakunitsuite/bunya/kenkou_iryou/iryouhoken/newpage_21054.html.

[8] Toyokuni E, Okada H, Hamaguchi M (2024). Eating behaviors and incidence of type 2 diabetes in Japanese people: the population-based Panasonic cohort study 15. J Diabetes Investig.

[9] Fukuma S, Iizuka T, Ikenoue T, Tsugawa Y (2020). Association of the National Health Guidance Intervention for Obesity and Cardiovascular Risks with health outcomes among Japanese men. JAMA Intern Med.

[10] Fukuma S, Mukaigawara M, Iizuka T, Tsugawa Y (2022). Impact of the national health guidance intervention for obesity and cardiovascular risks on healthcare utilisation and healthcare spending in working-age Japanese cohort: regression discontinuity design. BMJ Open.

[11] Lahelma E, Aittomäki A, Laaksonen M (2013). Cohort profile: the Helsinki Health Study. Int J Epidemiol.

[12] Marmot M, Brunner E (2005). Cohort profile: the Whitehall II study. Int J Epidemiol.

[13] Schmidt MI, Duncan BB, Mill JG (2015). Cohort profile: longitudinal study of adult health (ELSA-Brasil). Int J Epidemiol.

[14] Kitazawa A, Fukuda Y (2023). Sex-specific association of body mass index and fatty liver index with prevalence of renal hyperfiltration: a cross sectional study using Japanese health check-up data. BMC Nephrol.

[15] Kitazawa A, Maeda S, Fukuda Y (2021). Fatty liver index as a predictive marker for the development of diabetes: a retrospective cohort study using Japanese health check-up data. PLoS One.

[16] Sugimoto K, Yamada T, Kitazawa A, Fukuda Y (2024). Metabolic syndrome and depression: evidence from a cross-sectional study of real-world data in Japan. Environ Health Prev Med.

[17] (2025). Mutual Aid Association of Prefectural Government Personnel. https://www.chikyosai.or.jp.

[18] (2025). Ministry of Health, Labour, and Welfare. Standard Program of Health Checkup and Health Guidance. https://www.mhlw.go.jp/content/10900000/001231390.pdf.

[19] Iseki K, Konta T, Asahi K (2020). Impact of metabolic syndrome on the mortality rate among participants in a specific health check and guidance program in Japan. Intern Med.

[20] (2025). Ministry of Health, Labour and Welafe. NDB Open Data. https://www.mhlw.go.jp/stf/seisakunitsuite/bunya/0000177182.html.

[21] (2025). Ministry of Health, Labour and Welafe. Age-adjusted mortality rate by prefectures. https://www.mhlw.go.jp/toukei/list/nenchou.html.

[22] Yamaguchi M, Shimizu K, Nakazawa Y, Ikeda Y, Ohfusa H (2003). The relationship between occupations and lifestyle-related disease. Kenko Igaku.

[23] Ferraro RA, Leucker T, Martin SS, Banach M, Jones SR, Toth PP (2022). Contemporary management of dyslipidemia. Drugs.

[24] Väisänen D, Kallings LV, Andersson G, Wallin P, Hemmingsson E, Ekblom-Bak E (2020). Lifestyle-associated health risk indicators across a wide range of occupational groups: a cross-sectional analysis in 72,855 workers. BMC Public Health.

[25] Giskes K, Avendano M, Brug J, Kunst AE (2010). A systematic review of studies on socioeconomic inequalities in dietary intakes associated with weight gain and overweight/obesity conducted among European adults. Obes Rev.

[26] Gidlow C, Johnston LH, Crone D, Ellis N, James D (2006). A systematic review of the relationship between socio-economic position and physical activity. Health Educ J.

[27] Pampel FC, Krueger PM, Denney JT (2010). Socioeconomic disparities in health behaviors. Annu Rev Sociol.

[28] Wang T, Li Y, Zheng X (2023). Association of socioeconomic status with cardiovascular disease and cardiovascular risk factors: a systematic review and meta-analysis. Z Gesundh Wiss.

[29] (2025). Association of socioeconomic status with cardiovascular disease and cardiovascular risk factors: a systematic review and meta-analysis. Data relate to special health checkup and guideline. https://www.mhlw.go.jp/stf/newpage_03092.html.

[30] (2025). Ministry of Health, Labour and Welfare. 2016 National Survey of Health and Nurition. https://www.mhlw.go.jp/bunya/kenkou/eiyou/h28-houkoku.html.

